# Effects of Trait Anxiety on Error Processing and Post-error Adjustments: An Event-Related Potential Study With Stop-Signal Task

**DOI:** 10.3389/fnhum.2021.650838

**Published:** 2021-06-22

**Authors:** Meng-Tien Hsieh, Hsinjie Lu, Chia-I Lin, Tzu-Han Sun, Yi-Ru Chen, Chia-Hsiung Cheng

**Affiliations:** ^1^Department of Biomedical Sciences, Chang Gung University, Taoyuan, Taiwan; ^2^Laboratory of Brain Imaging and Neural Dynamics, Chang Gung University, Taoyuan, Taiwan; ^3^Department of Occupational Therapy and Graduate Institute of Behavioral Sciences, Chang Gung University, Taoyuan, Taiwan; ^4^Healthy Aging Research Center, Chang Gung University, Taoyuan, Taiwan; ^5^Department of Psychiatry, Chang Gung Memorial Hospital, Taoyuan, Taiwan

**Keywords:** trait anxiety, SSRT, ERN, Pe, post-error slowing, inhibitory control

## Abstract

The present study aimed to use event-related potentials with the stop-signal task to investigate the effects of trait anxiety on inhibitory control, error monitoring, and post-error adjustments. The stop-signal reaction time (SSRT) was used to evaluate the behavioral competence of inhibitory control. Electrophysiological signals of error-related negativity (ERN) and error positivity (Pe) were used to study error perception and error awareness, respectively. Post-error slowing (PES) was applied to examine the behavioral adjustments after making errors. The results showed that SSRT and PES did not differ significantly between individuals with high trait anxiety (HTA) and those with low trait anxiety (LTA). However, individuals with HTA demonstrated reduced ERN amplitudes and prolonged Pe latencies than those with LTA. Prolonged Pe latencies were also significantly associated with poorer post-error adjustments. In conclusion, HTA led to reduced cortical responses to error monitoring. Furthermore, inefficient conscious awareness of errors might lead to maladaptive post-error adjustments.

## Introduction

Trait anxiety refers to individuals' predisposition to respond to anxiety, worries, or fears to stressors and threats (McNally, 1989; Huang et al., 2012). Increasing evidence has shown that trait anxiety is associated with or predicts the prognosis of patients with an anxiety disorder. For example, Kang and colleagues reported that a higher level of trait anxiety predicts poorer health-related quality of life in patients with panic disorder (Kang et al., 2015). It has also been reported that trait anxiety is a predictor of the occurrence of post-traumatic stress disorder (PTSD) after experiencing an earthquake (Kadak et al., 2013) and of the severity of PTSD (Suliman et al., 2014). Despite compelling evidence showing abnormally high sensitivity to stress and threats in individuals with trait anxiety (Aitken et al., 1999; Berggren and Eimer, 2021), little is known about whether executive functioning is affected in this population. Inhibitory control and error monitoring are two key elements of executive function that substantially affect task performance in everyday life. Successful goal-directed tasks rely on efficiently suppressing irrelevant thoughts or behaviors to focus on task-relevant demands. Additionally, error detecting and monitoring can be used to adapt behaviors and improve task performance. Abnormalities in inhibitory control and error monitoring have been frequently reported in clinical forms of anxiety (Eysenck et al., 2007; Moser et al., 2013). However, the effects of trait anxiety, a subclinical form of anxiety, on inhibitory control and error monitoring remain inconclusive (Moser et al., 2012; Saunders and Inzlicht, 2020; Xia et al., 2020). Therefore, this study aimed to investigate whether and to what extent trait anxiety modulates inhibitory control, error processing, and adaptive behavior after making a mistake.

Many experimental tasks have been used to evaluate inhibitory functions, such as the go/no-go task, the Stroop task, and the stop-signal task (SST). Among them, the SST is designed to examine an individual's ability to cancel the initiated movement, and its neural correlates are relatively well-elucidated (Savostyanov et al., 2009; Neo et al., 2011; Sebastian et al., 2018). Furthermore, the SST conforms to the objectives of this study as it can be used to examine inhibitory control and error processing (Stahl and Gibbons, 2007; Li et al., 2008; Logan et al., 2014). The SST has been extensively used to measure the ability to stop initiated movements and to estimate the covert response-inhibition latency as the stop-signal reaction time (SSRT). Specifically, a longer SSRT indicates that an individual needs more time to inhibit their motor responses (Band et al., 2003; Verbruggen et al., 2008, 2019). However, no study has investigated the effects of trait anxiety on the SSRT. Thus, the first aim of the present study was to clarify how trait anxiety modulates inhibitory control as indexed by the SSRT.

Error-related negativity (ERN) and error positivity (Pe) are error-related electrophysiological indicators of performance monitoring and error detection. ERN is a salient negative deflection of the event-related potential (ERP) component, which shows maximum amplitude in fronto-central sites ~50 ms after committing an error (Chang et al., 2010; Gehring et al., 2018; Riesel, 2019). ERN is related to the processing of error detection, while Pe, a positive deflection of the ERP in centro-parietal sites that immediately follows ERN, is associated with error awareness (Nieuwenhuis et al., 2001; O'Connell et al., 2007; Santesso et al., 2011). Larger ERN amplitudes have been reported in higher vs. lower levels of trait anxiety (Hajcak et al., 2003a; Riesel et al., 2017); however, one study reported no significant difference in ERN amplitudes between individuals with higher (HTA) and lower trait anxiety (LTA) (Aarts and Pourtois, 2010). Although most studies suggest that trait anxiety does not modulate Pe activity (Aarts and Pourtois, 2010; Wu et al., 2019), Hajcak and colleagues reported that reduced Pe amplitudes are found in individuals with high levels of negative affect (Hajcak et al., 2004). Taken together, it remains obscure whether trait anxiety affects ERN and Pe. Thus, the second aim of the present study was to examine how trait anxiety modulates ERN and Pe activities by comparing these two components between individuals with HTA and LTA.

Post-error slowing (PES), the phenomenon of the slowing down of motor responses after making a mistake (i.e., prolonged reaction time after committing an error), is a common index of adaptive behavior to errors (Hajcak et al., 2003b; Danielmeier and Ullsperger, 2011; Ullsperger and Danielmeier, 2016). A few studies have examined how trait anxiety modulates PES. One study indicated no significant difference in PES between HTA and LTA using the go/no-go task (Aarts and Pourtois, 2010). Furthermore, it has been suggested that PES does not interact with trait anxiety on the Simon task (Van der Borght et al., 2016). However, it is unknown whether different tasks affect PES in individuals with HTA and LTA. Thus, the third aim of the present study was to examine the effects of trait anxiety on PES using the SST.

In addition to objective indicators, such as the SSRT and PES, the present study also assessed the self-evaluation of cognitive function, which was acquired through the Cognitive Failure Questionnaire (CFQ) (Broadbent et al., 1982). The CFQ has been used to evaluate subjective perception of cognitive failure during daily life. A positive correlation between trait anxiety and the CFQ score has been documented (Righi et al., 2009), suggesting that individuals with HTA self-report more cognitive failures. However, the relationship between the CFQ and error processing is unclear.

The main purposes of the present study are three-fold. Firstly, we compared the SSRT, a behavioral index of inhibitory control, between the HTA and LTA groups. Secondly, we examined the differences in the ERN and Pe responses between the HTA and LTA groups. Finally, we compared PES between the HTA and LTA groups to evaluate whether trait anxiety affects the performance of post-error adjustments. Furthermore, based on ERPs with significant between-group differences, we tested whether electrophysiological signatures of error processing (i.e., ERN and Pe) would be associated with behavioral post-error adjustment (i.e., PES) and self-reported cognitive performance (i.e., CFQ).

## Methods

### Participants

A total of 400 undergraduate students completed the State-Trait Anxiety Inventory (STAI) and the CFQ. The STAI consists of the self-reported state STAI (STAI-S) for immediate levels of anxiety and the trait STAI (STAI-T) for levels of “general anxiety” (Spielberger and Vagg, 1984). The 25-item CFQ has a five-point scale (0 = never, 1 = very rare, 2 = occasionally, 3 = quite often, and 4 = very often) and was used to measure self-reported cognitive performance on daily tasks requiring different kinds of attention and memory ability, such as “Do you find you forget whether you've turned off a light or a fire or locked the door?” “Do you leave important letters unanswered for days?” “Do you have troubles making up your mind?” and “Do you find you forget what you came to the shops to buy?” Among the 400 participants, 20 from the top 10% and 20 from the bottom 10% of the STAI-T score distribution comprised the HTA and LTA groups, respectively. These 40 subjects, who finally participated in the ERP study, confirmed that they were right-handed and free from substance addiction and a history of neurological/psychiatric disorders by self-report. They had normal or corrected-to-normal vision.

This study was approved by the Institutional Review Board of Chang Gung Memorial Hospital (Linkou, Taiwan) and was performed following approved guidelines and regulations. Written informed consent was obtained from each participant after a detailed description of the experimental procedures was provided.

### Experimental Procedures

**Figure 1:**
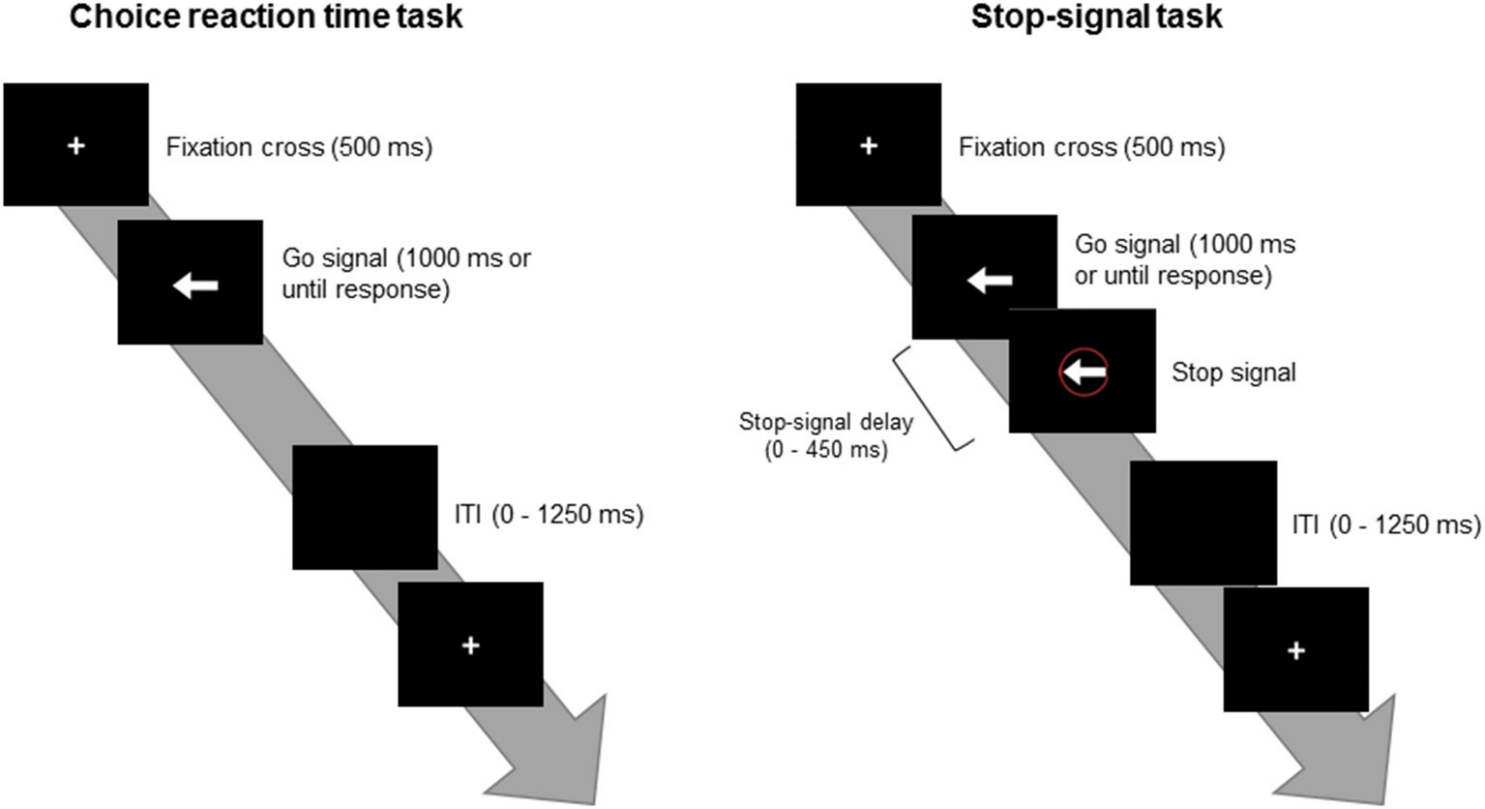
Depictions of the choice reaction time task (*left*) and the stop-signal task (*right*). *ITI*, inter-trial interval.

Figure 1 depicts the SST procedure. The choice reaction task consisted of 120 trials, in which white arrows were randomly displayed pointing to the left (*p* = 50%) or to the right (*p* = 50%). These stimuli were presented on a black background after a 500-ms white crosshair was presented in the center of the monitor and disappeared after the subject responded or 1,000 ms had passed. All participants were instructed to press the “F” key on the keyboard with their left index finger when they see an arrow pointing to the left and to press the “J” key with their right index finger when they see an arrow pointing to the right. The participants were instructed to respond to the stimuli as quickly and correctly as possible. The inter-trial interval was random between 0 and 1,250 ms (average = 625 ms). The average reaction time (RT) and the standard deviation (SD) for each participant were recorded to determine their RT + 2 SD time as the limit of the response time on the subsequent SST to avoid the subject taking a strategic delay, which would lead to an improved inhibitory success rate to the Stop trial. A short practice consisting of 20 trials was delivered to each subject prior to the formal task.

The SST consisted of 360 frequent Go trials (*p* = 75%) and 120 infrequent Stop trials (*p* = 25%). The Go trials were presented with arrows randomly pointing to the left (180 trials, *p* = 50%) or the right (180 trials, *p* = 50%). The infrequent Stop stimuli comprised red circles around the arrows that appeared several hundreds of milliseconds immediately after the Go stimuli. All participants were instructed to inhibit the response to the Go stimuli when they see the Stop signal. The SST was designed for an accuracy rate of about 50% on the Stop trials for each participant. In this case, the stop-signal delay (SSD), the time interval between the Go and Stop trials, was adjusted according to a participant's behavioral performance. The initial SSD was set to 250 ms with steps of 50 ms. If the participant failed to suppress the response to the Stop trials, the SSD was decreased 50 ms in steps until it reaches 0 ms. In contrast, if the participant successfully inhibited the response to the Stop trial, 50 ms was added in steps until the SSD reaches 450 ms. In addition, if a participant provided an incorrect response or exceeded the response time limit, a warning sound was delivered. Following 24 practice trials, all participants underwent four blocks of the SST, consisting of 120 trials per block.

### Electrophysiological Recordings and Data Pre-processing

ERPs were recorded during the SST using a 34-channel elastic cap (EasyCap GmbH, Herrsching, Germany) with Ag/AgCl electrodes according to the international 10–20 system. Eye blinks and eye movements were recorded by four vertical and horizontal electrooculograms. Scalp electroencephalogram (EEG) electrode impedance was <5 kΩ. A 40-channel QuickAmp amplifier system and Vision Recorder software (Brain Products GmbH, Gilching, Germany) were used for data acquisition, with a sampling rate of 1,000 Hz. The ERP data were off-line re-referenced to the average of the two mastoid electrodes and filtered with a bandpass of 0.1–30 Hz. Response-locked epochs from 250 ms before the motor response to 750 ms after response onset were analyzed. The 200-ms interval from −250 to −50 ms before the response onset served as the baseline. Epochs contaminated by ocular artifacts or EEG signals exceeding ±60 μV were excluded from further analysis. The epochs were averaged separately for three response trial types: correct responses to the Go trials (CG), successful responses to the Stop trials (SS), and unsuccessful responses to the Stop trials (US).

### Analysis of the SSRT and PES Behavioral Parameters

The SSRT, calculated as the “CG–SSD” RT, was defined as the time for the subject to successfully inhibit responses to the Stop trial (Verbruggen et al., 2008, 2019). The PES, measured by the RT of [“CG following US”—“CG preceding US”], was defined as the adaptive slowing of responses after making an error (Dutilh et al., 2012).

### ERN and Pe Analysis

The ERP components of interest were calculated by subtracting the responses to CG from those of US (i.e., ΔERN and ΔPe). The ΔERN component was analyzed at the FCz electrode (O'Connell et al., 2007; Chang et al., 2010) and the Pe component analyzed at the Pz electrode (Falkenstein et al., 2000; Schroder et al., 2020). The peak latency of ΔERN was defined as the time when maximal activity occurred between −50 and 150 ms; the mean ΔERN amplitude was defined as the average amplitude between the ±20-ms time window centered on the peak amplitude (Clayson et al., 2013; Riesel et al., 2017; Beatty et al., 2018). The ΔPe peak latency was defined as the time of maximal activity between 150 and 400 ms; the ΔPe mean amplitude was defined as the average amplitude between the ±50-ms time window centered on the peak amplitude (Clayson et al., 2013; Beatty et al., 2018).

### Statistical Analysis

All data are presented as the mean ± SD. Two-way mixed-design ANOVAs were used to determine the effects of trait anxiety (between-subject factors: HTA and LTA) and trial type (within-subject factors: CG and US) on RT. To avoid the influence of outliers (values above and below the mean ± 2 SD in each group), seven data records were removed from the statistical analysis (HTA group: one mean ΔERN amplitude record, two ΔERN peak latency records, and one ΔPe peak latency record; LTA group: one ΔERN peak latency record and two ΔPe peak latency records). A non-parametric analysis was performed as the ΔPe peak latencies were not normally distributed after excluding the outliers. The Mann–Whitney *U*-test was used to compare the behavioral, ERP, and self-reported CFQ data between the HTA and LTA groups. Based on the ERP results showing significant between-group differences, if recorded, the partial correlations of the ERPs with the behavioral data and the CFQ scores were evaluated with age and gender as covariates. A two-tailed *p*-value < 0.05 was considered significant.

The effect sizes (Cohen's *d* or partial eta squared, as appropriate) for each comparison were also calculated. Cohen's *d* effect sizes between 0.2 and 0.5 were considered small, those between 0.5 and 0.8 were considered moderate, and those over 0.8 were considered large (Cohen, 1992).

## Results

Table 1 lists the demographic and behavioral data of the HTA and LTA groups. The means of the age and gender distributions did not differ significantly between the two groups. Individuals with HTA also showed significantly elevated state anxiety compared to those with LTA (*p* < 0.001, Cohen's *d* = 2.125). In addition, individuals with HTA self-reported more failed daily cognitive tasks than those with LTA (*p* < 0.001, Cohen's *d* = 2.491). The accuracy rates on the Go trials did not significantly differ between the HTA and LTA groups (*p* = 0.170, Cohen's *d* = 0.661), suggesting that both groups performed this task with similar levels of attention. Consistent with previous concepts (Logan et al., 2014), our results show that the RT of US was significantly shorter than that of CG (US = 410.6 ± 52.7 ms, CG = 475.97 ± 71.06 ms, *p* < 0.001, $\eta_{\text{p}}^{2}$ = 0.864). However, neither a main effect of group (HTA = 448.88 ± 74.48 ms, LTA = 437.69 ± 66.38 ms, *p* = 0.569, $\eta_{\text{p}}^{2}$ = 0.009) nor an interactive effect (*p* = 0.62, $\eta_{\text{p}}^{2}$ = 0.007) on RT was detected.

**Table 1:** Demographic and behavioral data (mean ± SD) in subjects with higher (HTA) and lower trait anxiety (LTA).

	**HTA** ** (*n* = 20)**	**LTA** ** (*n* = 20)**	***p*-value**	**Cohen's *d***
Age (years)	21.2 ± 1.44	21.95 ± 3.22	0.765	–
Gender (male/female)	4/16	8/12	0.168	–
STAI-T	62.45 ± 4.71	26.1 ± 3.04	<0.001	9.170
STAI-S	44.25 ± 8.45	28.00 ± 6.75	<0.001	2.125
CFQ	53.85 ± 15.11	22.45 ± 9.46	<0.001	2.491
Go RT (ms)	482.63 ± 75.37	469.32 ± 67.43	0.626	0.186
Unsuccessful stop RT (ms)	415.14 ± 57.53	406.05 ± 48.46	0.646	0.171
Go accuracy (%)	96.46 ± 3.68	98.32 ± 1.51	0.170	0.661
Stop accuracy (%)	48.88 ± 1.46	49.54 ± 2.63	0.168	0.310
SSRT (ms)	316.75 ± 32.94	299.78 ± 34.45	0.083	0.504
PES (ms)	19.89 ± 42.78	13.55 ± 28.34	0.344	0.175

*SD, standard deviation; STAI-T, State-Trait Anxiety Inventory-Trait; STAI-S, State-Trait Anxiety Inventory-State; CFQ, Cognitive Failure Questionnaire; RT, reaction time; SSRT, stop-signal reaction time; PES, post-error slowing*.

The first aim of the present study was to investigate whether SSRT was affected by trait anxiety. The results showed a trend for a difference (*Z* = −1.731, *p* = 0.083, Cohen's *d* = 0.504) in the SSRT between the HTA (316.75 ± 32.94 ms) and LTA (299.78 ± 34.45 ms) groups.

**Figure 2:**
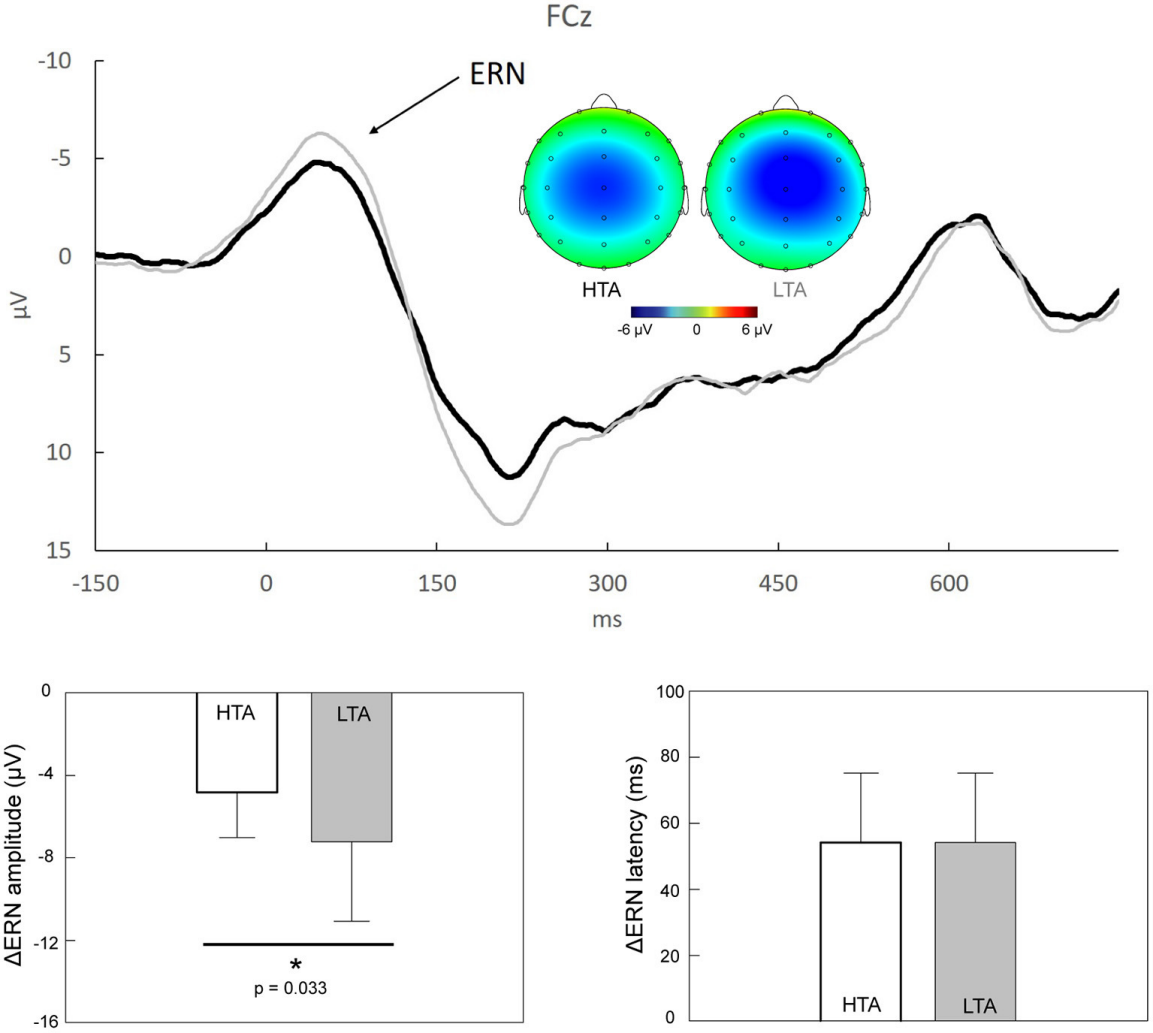
*Upper panel*: Response-locked grand average ΔERN (i.e., “error” minus “correct”) at FCz in individuals with high trait anxiety (HTA, *black trace*) and those with low trait anxiety (LTA, *gray trace*). The scalp topographies representing the peaks of ΔERN in the HTA (23–63 ms) and LTA (26–66 ms) groups are also illustrated. *Lower panel*: Statistical results showed that, compared to individuals with LTA, those with HTA demonstrated a significant reduction of ΔERN amplitudes. *p < 0.05.

The second aim of the present study was to investigate whether ΔERN and ΔPe differed between the HTA and LTA groups. Figure 2 shows that prominent ΔERN components, peaking 0–100 ms after the onset of the response, were clearly observed in the HTA and LTA groups. The topographic maps demonstrated a fronto-central distribution of the ΔERN activities. Individuals with HTA (−4.83 ± 2.21 μV) exhibited significantly reduced ΔERN mean amplitudes compared to individuals with LTA (−7.23 ± 3.88 μV; *Z* = −2.135, *p* = 0.033, Cohen's *d* = 0.755). No significant between-group difference was observed in the ΔERN peak latencies (*Z* = −243, *p* = 0.808, Cohen's *d* = 0.002).

**Figure 3:**
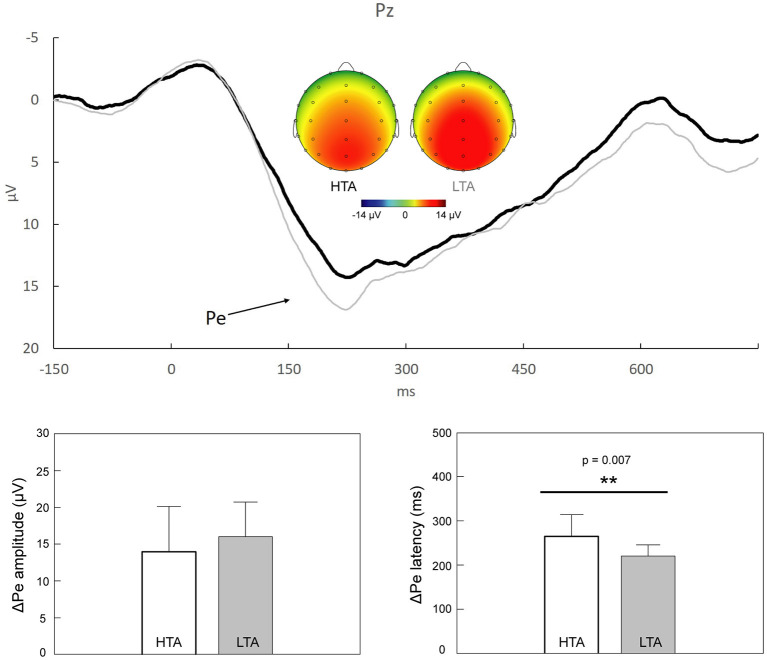
*Upper panel*: Response-locked grand average ΔPe (i.e., “error” minus “correct”) at Pz in individuals with high trait anxiety (HTA, *black trace*) and those with low trait anxiety (LTA, *gray trace*). The scalp topographies representing the peaks of ΔPe in the HTA (174–274 ms) and LTA (173–273 ms) groups are also illustrated. *Lower panel*: Statistical results showed that, compared to individuals with LTA, those with HTA demonstrated a significant delay of ΔPe latencies. **p < 0.01.

Figure 3 shows that the grand average ΔPe components peaked 150–300 ms after the onset of the response, with a topographically centro-parietal distribution. Individuals with HTA (264.26 ± 50.25 ms) exhibited prolonged ΔPe peak latencies compared to those with LTA (220.33 ± 24.86 ms; *Z* = −2.69, *p* = 0.007, Cohen's *d* = 1.099). No significant between-group difference was observed in the ΔPe mean amplitudes (*Z* = −1.055, *p* = 0.291, Cohen's *d* = 0.373).

**Figure 4:**
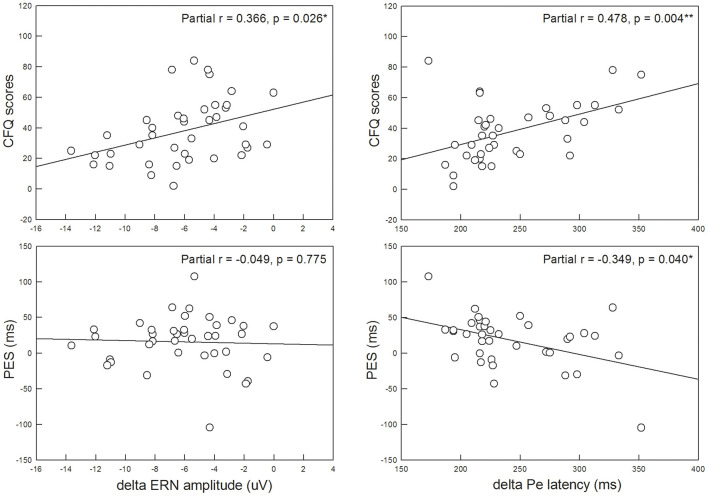
The Cognitive Failure Questionnaire (CFQ) score was significantly associated with the ΔERN mean amplitude and ΔPe peak latency, suggesting that more self-reported failures in cognitive tasks are related to more reduced efficiency in error monitoring. Post-error slowing (PES) was also significantly associated with the ΔPe peak latency, suggesting that poorer adaptive post-error adjustments are related to more deficiency in error awareness. *p < 0.05; **p < 0.01.

The third aim of the present study was to assess whether trait anxiety affected post-error adjustments. Our results showed that the PES did not significantly differ between the HTA (19.89 ± 42.78 ms) and LTA (13.55 ± 28.34 ms) groups (*Z* = −0.947, *p* = 0.344, Cohen's *d* = 0.175). However, PES was significantly and negatively correlated with the ΔPe peak latency (partial *r* = −0.349, *p* = 0.040), suggesting that a greater deficiency in error awareness was associated with poorer adaptive post-error adjustments (i.e., less slowing of RT after errors). Furthermore, a higher CFQ score was significantly and positively correlated with more attenuated ΔERN mean amplitudes (partial *r* = 0.366, *p* = 0.026) and more delayed ΔPe peak latencies (partial *r* = 0.478, *p* = 0.004), suggesting that reduced efficiency in error monitoring was concomitant with more self-reported failures in daily cognitive tasks (Figure 4).

## Discussion

The present study investigated the effects of trait anxiety on error processing and post-error adjustments using ERP recordings with the SST. The behavioral data showed that the SSRT and PES did not differ significantly between the HTA and LTA groups. However, the ERP results revealed that individuals with HTA demonstrated reduced ΔERN amplitudes and prolonged ΔPe latencies than those with LTA. The correlational results showed that the ΔERN amplitudes and ΔPe latencies were significantly correlated with the CFQ score. Notably, the ΔPe latencies were significantly correlated with PES.

Our first finding revealed no significant difference in the SSRT between the HTA and LTA groups, suggesting that trait anxiety does not have a substantial effect on behavioral competence of response inhibition. However, other studies that have applied the go/no-go (Xia et al., 2020) or the Stroop task (Basten et al., 2011) revealed behavioral deficits of inhibitory function in individuals with HTA compared to those with LTA. It should be noted that the *p*-value (*p* = 0.08, two-tailed) in the present study was very close to significance given a sample size with 20 subjects in each group. The effect size was calculated to further verify our data. The results showed that Cohen's *d* was 0.504, considered a medium effect size in the comparison between the HTA and LTA groups. Another plausible reason is that there might be a psychopathological threshold in the anxiety level to provoke behavioral impairments in response inhibition. Thus, future research should explore the effects of trait anxiety on the SSRT using a larger sample size and the maximum difference in the STAI-T scores between the HTA and LTA groups.

However, higher ΔERN amplitudes and shortened ΔPe latencies were found in the LTA compared to the HTA group, suggesting that individuals with LTA detected errors more quickly and more efficiently. The available literature that has investigated the effects of trait anxiety on ERN have reported mixed results. For example, Hajcak and colleagues reported that healthy volunteers with higher scores on the Penn State Worry Questionnaire (PSWQ) showed enhanced ERN amplitudes relative to non-anxious subjects (Hajcak et al., 2003a). Similarly, Riesel and colleagues, who used the Illness-Attitude Scale and the Whiteley Index (WI), revealed that subjects with healthy anxiety have augmented ERN amplitudes compared to healthy controls (Riesel et al., 2017). In contrast, a study that used the STAI-T to divide healthy participants into HTA and LTA groups found no significant difference in the ERN amplitudes between these two groups (Aarts and Pourtois, 2010). More importantly, a recent meta-analysis, which comprehensively investigated the relationship between anxiety and ERN responses, reported that subclinical anxiety (e.g., healthy individuals with higher scores on anxiety scales) was not significantly associated with ERN amplitude (Saunders and Inzlicht, 2020). The first reason to account for the aforementioned discrepancies is that different aspects of anxiety were measured in different studies. For example, items in the PSWQ and WI are more related to obsessive–compulsive disorder or hypochondriasis, respectively, whereas the STAI helps clinical practitioners distinguish depression and anxiety. The second cause is potentially the different methodologies used. Some studies applied flanker tasks (Riesel et al., 2013, 2017; Schroder et al., 2020), while others used the go/no-go task or the SST (Falkenstein et al., 2000; Aarts and Pourtois, 2010; Reinhart et al., 2012). Different cognitive processes (e.g., working memory in the go/no-go task or conflict monitoring in the flanker task) may have dynamic interactions in error detection when a participant makes a mistake (Riesel et al., 2013). Although the relationship between ERN and clinical anxiety is well-defined, our present results provide insights for future studies to comprehensively investigate different kinds of trait anxiety (e.g., anxious apprehension/distress and anxious arousal/fear) on the ERN. Furthermore, due to the limited literature on Pe in trait anxiety, replication of our present work with a larger sample size is imperative.

The present study did not find significant differences in PES between the HTA and LTA groups, which agrees with previous investigations on PES either using the go/no-go task (Aarts and Pourtois, 2010) or the Simon task (Van der Borght et al., 2016). Taken together with previous findings, our results suggest that trait anxiety did not have a profound influence on an individual's behavioral performance of post-error adjustments. However, when pooling all of the subjects together, PES was significantly associated with ΔPe peak latency, but not with ΔERN. Previous studies have reported larger Pe responses to perceived than unperceived errors; however, such a difference is not observed in ERN (Nieuwenhuis et al., 2001; O'Connell et al., 2007). Furthermore, larger Pe amplitudes have been reported to be associated with more PES on a flanker task (Schroder et al., 2020), suggesting that more conscious awareness of errors is concomitant with more RT after making an error. Consistent with these findings, our study used the SST and supported that increased error awareness (i.e., shortened ΔPe peak latency) could lead to more adjustments to compensate for mistakes (i.e., longer PES). The specificity of the present association for ΔPe, but not for ΔERN, is also noteworthy. Consistent with our present results, several studies have found no association between ERN and PES in worried students (Hajcak and Simons, 2002; Hajcak et al., 2003a). However, others have detected such a relationship (West and Travers, 2008; Overbye et al., 2019). Taken together, although our data suggest that higher neural responses to error awareness are related to more adaptive adjustments after errors, this result should be carefully interpreted based on the relatively small correlation coefficient (partial *r* = −0.349).

In terms of the CFQ, previous studies have indicated that subjects with a higher frequency of cognitive failure are associated with higher levels of state and trait anxiety, suggesting that trait anxiety affects self-reported cognitive failure (Righi et al., 2009). Therefore, we further examined whether the CFQ was associated with brain responses related to error monitoring. Our data showed that the CFQ score was positively correlated to the ΔERN mean amplitude and ΔPe peak latency, suggesting that more self-perceived cognitive dysfunction is correlated with reduced error perception and more time to be aware of errors.

Several limitations and methodological considerations of this study should be addressed. Firstly, the relatively small sample size may have decreased statistical power in the SSRT comparison between the HTA and LTA groups. Secondly, we applied stringent criteria (i.e., values above or below the mean ± 2 SD) to define the outliers. With a total of 160 records (i.e., ΔERN mean amplitudes, ΔERN peak latencies, ΔPe mean amplitudes, and ΔPe peak latencies), only seven records were removed from statistical analyses. Notably, the ΔPe peak latencies were still not normally distributed after removing these outliers. Therefore, we used a more stringent threshold to avoid the influence of extreme values on the ERP grand average data and statistical results. Finally, all subjects who participated in this study were undergraduate and graduate students; thus, the results may not be generalizable to other age groups.

This study, to the best of our knowledge, is the first to simultaneously investigate how trait anxiety affects inhibitory control, error processing, and post-error adjustments on the SST. Despite no significant between-group difference in behavioral performance of inhibitory control and post-error adjustments, individuals with higher levels of trait anxiety showed reduced cortical efficiency in error monitoring. The correlational results further highlighted that subjects with more error awareness may make more adjustments to compensate for mistakes.

## Data Availability Statement

The raw data supporting the conclusions of this article will be made available by the authors, without undue reservation.

## Ethics Statement

The studies involving human participants were reviewed and approved by Institutional Review Board of Chang Gung Memorial Hospital. The patients/participants provided their written informed consent to participate in this study.

## Author Contributions

C-HC conceived and designed the work. M-TH, HL, C-IL, T-HS, and Y-RC acquired the data. M-TH, HL, C-IL, T-HS, and Y-RC analyzed the data. M-TH, HL, C-IL, T-HS, Y-RC, and C-HC participated in the discussion and provided the comments. M-TH, HL, and C-HC wrote the paper. All of the authors have read and approved the manuscript.

## Conflict of Interest

The authors declare that the research was conducted in the absence of any commercial or financial relationships that could be construed as a potential conflict of interest.
